# Microarray Analysis of Gene Expression by Skeletal Muscle of Three Mouse Models of Kennedy Disease/Spinal Bulbar Muscular Atrophy

**DOI:** 10.1371/journal.pone.0012922

**Published:** 2010-09-23

**Authors:** Kaiguo Mo, Zak Razak, Pengcheng Rao, Zhigang Yu, Hiroaki Adachi, Masahisa Katsuno, Gen Sobue, Andrew P. Lieberman, J. Timothy Westwood, D. Ashley Monks

**Affiliations:** 1 Department of Psychology, University of Toronto at Mississauga, Mississauga, Ontario, Canada; 2 Department of Cell and Systems Biology, University of Toronto, Mississauga, Ontario, Canada; 3 Department of Pathology, University of Michigan Medical School, Ann Arbor, Michigan, United States of America; 4 Department of Neurology, Nagoya University Graduate School of Medicine, Nagoya, Japan; 5 Institute of Advanced Research, Nagoya University, Nagoya, Japan; 6 Institute of Medical Science, University of Toronto, Mississauga, Ontario, Canada; University of Cambridge, United Kingdom

## Abstract

**Background:**

Emerging evidence implicates altered gene expression within skeletal muscle in the pathogenesis of Kennedy disease/spinal bulbar muscular atrophy (KD/SBMA). We therefore broadly characterized gene expression in skeletal muscle of three independently generated mouse models of this disease. The mouse models included a polyglutamine expanded (polyQ) AR knock-in model (AR113Q), a polyQ AR transgenic model (AR97Q), and a transgenic mouse that overexpresses wild type AR solely in skeletal muscle (HSA-AR). HSA-AR mice were included because they substantially reproduce the KD/SBMA phenotype despite the absence of polyQ AR.

**Methodology/Principal Findings:**

We performed microarray analysis of lower hindlimb muscles taken from these three models relative to wild type controls using high density oligonucleotide arrays. All microarray comparisons were made with at least 3 animals in each condition, and only those genes having at least 2-fold difference and whose coefficient of variance was less than 100% were considered to be differentially expressed. When considered globally, there was a similar overlap in gene changes between the 3 models: 19% between HSA-AR and AR97Q, 21% between AR97Q and AR113Q, and 17% between HSA-AR and AR113Q, with 8% shared by all models. Several patterns of gene expression relevant to the disease process were observed. Notably, patterns of gene expression typical of loss of AR function were observed in all three models, as were alterations in genes involved in cell adhesion, energy balance, muscle atrophy and myogenesis. We additionally measured changes similar to those observed in skeletal muscle of a mouse model of Huntington's Disease, and to those common to muscle atrophy from diverse causes.

**Conclusions/Significance:**

By comparing patterns of gene expression in three independent models of KD/SBMA, we have been able to identify candidate genes that might mediate the core myogenic features of KD/SBMA.

## Introduction

Kennedy disease/spinal bulbar muscular atrophy (KD/SBMA), is a progressive neuromuscular disease [1], [2], which is caused by an expanded trinucleotide repeat length encoding the polyglutamine (polyQ) tract of the androgen receptor (AR) gene [3]. Polyglutamine expansion of other genes similarly results in neurodegenerative conditions, such as Huntington's disease and several autosomal dominant spinocerebellar ataxias [4]. The mechanisms that mediate neurodegeneration in the polyQ diseases remain poorly understood. The orthodox view of KD/SBMA is that the polyQ expanded AR has a toxic gain of function and also a loss of trophic function within motoneurons, resulting in neuron loss and consequently denervation-induced muscular atrophy. However, recent insight from mouse models of KD/SBMA have suggested that primary myogenic pathology, and more specifically pathological alterations in gene expression within skeletal muscle, might also contribute to this disease (reviewed in [5]). This evidence consists mainly of 2 major findings: that myopathy precedes neuropathy in mice with genetic polyQ expansion mutations in the AR [6], and also that overexpression of AR in muscle fibers is sufficient to reproduce several hallmark features of KD/SBMA [7]. Additional circumstantial evidence includes the presence of myopathy and transcriptional alterations in polyQ expanded AR mutant mice [6], [7], [8], [9], and studies of human pathology [6], [10], [11].

Transcriptional disturbance caused by polyQ expansion is thought to contribute to the etiology of KD/SBMA. Loss of the normal transcriptional functions of AR [12], [13], is paired with gain of toxic function, in which novel interactions of AR with cofactors results in aberrant transcriptional activation and/or repression [14]. An additional possibility is that transcriptional disturbance might occur indirectly, due to pathology at the cellular, tissue, organ or system level [5]. For example, muscle from polyQ AR mutant mice exhibit alterations in gene expression typical of denervation [6], [7], [15], which seems likely to originate as a cell non-autonomous response to neuropathy. Other candidate genes which might mediate pathology in KD/SBMA have been identified, including vascular endothelial growth factor (*Vegfa*) [7], [9], P300/CREB binding protein (*P300/CBP*) [9], [16], heat shock proteins [17], [18], [19], [20], [21], [22], [23], [24], and genes related to mitochondrial function [25]. More systematic studies of gene expression have been performed on cell lines [14], but to date, there have been no published reports of transcriptional profiling of mouse models of KD/SBMA.

Research into the etiology of KD/SBMA has been hampered somewhat by mouse models being analyzed individually, with little or no direct comparison between models. We therefore included several mouse models that differ significantly in the type of induced mutation and in some features of their phenotype (reviewed in [5]), but share common pathological features, including: myopathy, neuropathy, sex biased neuromuscular atrophy, ligand-dependant pathology, and transcriptional disturbance. HSA-AR mice, which lack polyQ AR despite having these features of KD/SBMA pathology, were included in the comparison to allow us to evaluate those alterations which are unique to polyQ AR. Additionally, we compared these results with published microarray analysis of gene expression in skeletal muscle of mice with related conditions, such as AR knock out (ARKO, [26]), DHT treatment of mice [27], [28], Huntington's disease [29], denervation induced and chronic muscle wasting [15], [30] to gain insight into the underlying systemic, cellular and molecular pathological mechanisms.

## Results

**Figure 1:**
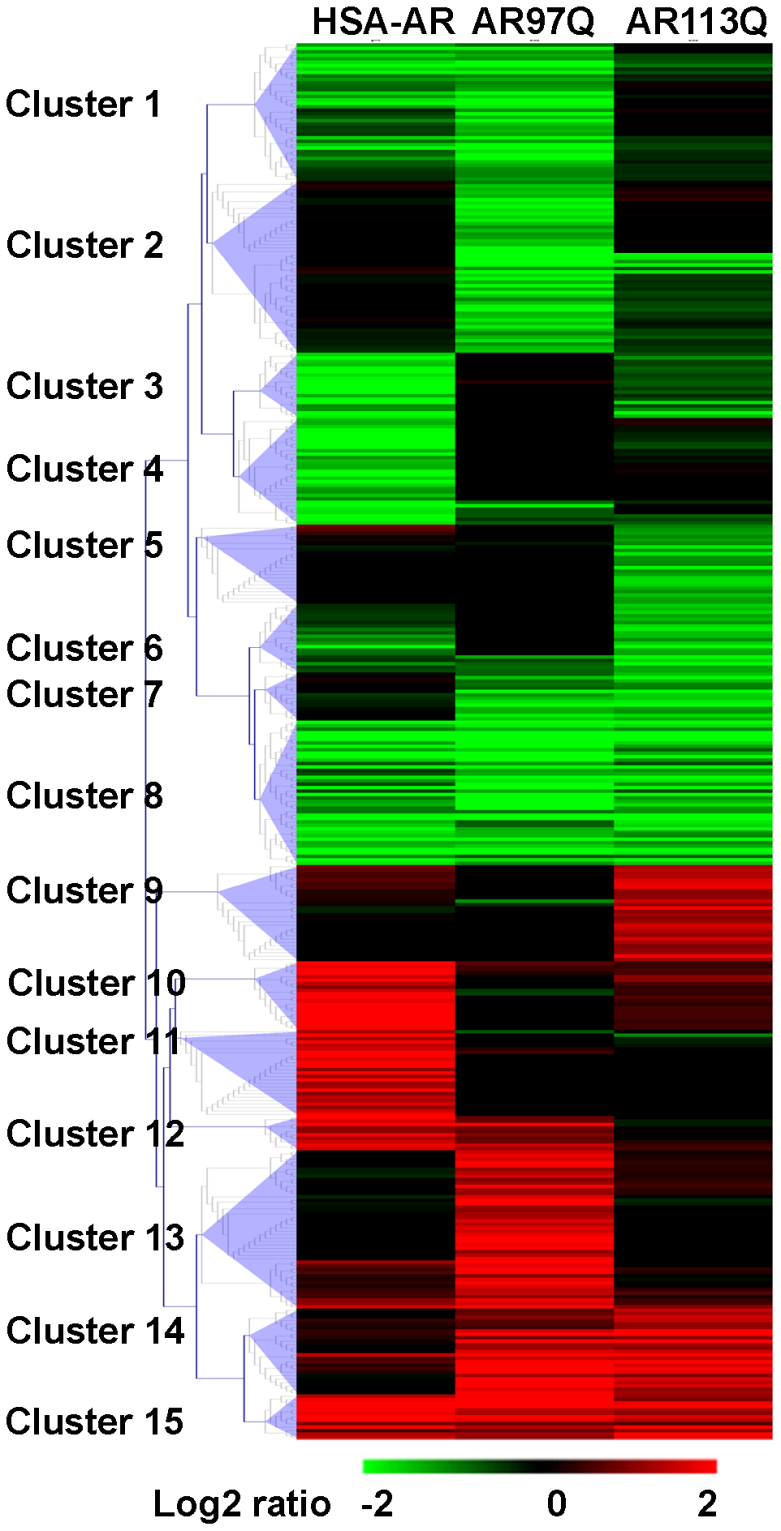
Venn diagrams of microarray results by mouse model. Diagram representing the number of genes whose expression differed from WT controls for each mutant strain (HSA-AR, AR97Q, AR113Q), and the number of such genes shared by each model. Notably, the overlap between HSA-AR and either of the polyQ models was similar to the overlap between the polyQ AR samples. Gene numbers were obtained by examining gene lists generated by a 2-fold, p≤0.05 criterion.

Complete lists of differentially regulated genes resulting from microarray analysis of HSA-AR, AR97Q and AR113Q are presented in the supporting material (Tables S1, S2, S3). The degree to which genes are regulated similarly between these models is represented by Venn diagram (Figure 1). This analysis indicated a similar degree of overlap between the 3 models, and suggested that a core pattern of altered gene expression is associated with the symptoms of KD/SBMA common to the three models. The list of genes in the intersection between the 3 models is reported in Table S4.

The list of candidate genes presented in Table S4 notably includes several whose functions relate to differentiation and/or atrophy of muscle. These include: DNA-damage-inducible transcript 4-like (*Ddit4l*), Enabled homolog (*Enah*), F box protein 32 (*Fbxo32*), and Integrin beta 1 binding protein 3 (*Itgb1bp3*). Other differentially regulated genes are implicated in regulating oxidative metabolism within skeletal muscle including: *Ddit4l*, Phospholipase A2, group VII (*Pla2g7*), and Phosphorylase kinase alpha 1 (*Phka1*). *Ddit4l*, also known as REDD2 and SMHS1, inhibits muscle growth via the *Igf1*/mTOR pathway and is regulated during atrophy of rat soleus muscle and the associated fiber type transition from oxidative to glycolytic [31], [32]. *Enah*, also known as MENA, modulates the actin cytoskeleton and cell signaling along with VASP. Dominant negative inhibition of both *Enah* and VASP results in cardiomyopathy [33]. *Itgb1bp3*, also known as MIBP, is developmentally regulated during myogenic differentiation and is thought to inhibits myocyte adhesion to laminin and myocyte production of laminin [34], [35]. Allelic variants of *Pla2g7* can promote reductions in adiposity following exercise in human populations [36], and reduction of adipose tissue is a known function of myocyte AR [37]. Allelic variants in *Phka1* in human populations are associated with metabolic myopathy [38], [39]. When considered in the context of the putative protective or toxic functions of these genes, the general pattern of regulation observed in our samples for *Ddit4l*, *Enah* and *Itgb1bp3* are consistent with adaptive tissue response to toxicity and that observed for *Phka1* and *Pla2g7* is consistent with a causal role in myopathy.

*Fbxo32* deserves special attention, as it is thought to activate a pattern of gene expression which is associated with diverse forms of muscle wasting [15], [30], [40]. Because muscle wasting is a cardinal feature of KD/SBMA and wasting is present in all 3 models, we were not surprised to find *Fbxo32* in all of the gene lists (Table S4 and Table 1) However, *Fbxo32* was unexpectedly *decreased* in all 3 models, whereas increases in *Fbxo32* were observed in other cases of muscle wasting, including diabetes, renal failure, cancer and denervation [15], [30]. Nonetheless, genes thought to be downstream from *Fbxo32* were also found to be affected in a manner consistent with studies of muscle atrophy (Table S4 and S5), suggesting a non-canonical activation of this pathway. In other words, these results suggest that muscle atrophy in KD/SBMA may not be not triggered by increases in *Fbxo32*, but rather that genes downstream of this ubiquitin ligase may mediate atrophy.

**Table 1:** Validation of the results of microarray experiments by qRT-PCR analysis.

	Microarray Fold Change Relative to WT			qRT-PCR Fold Change Relative to WT (P-value)		
Unigene Symbol	HSA-AR	AR97Q	AR113Q	HSA-AR	AR97Q	AR113Q
*Vegfa*	−1.86	−5.11	−2.22	−1.74 (0.01)	−2.35 (0.03)	−1.92 (0.02)
*Fbxo32*	−3.46	−6.69	−6.67	−2.56 (0.03)	−2.78 (0.05)	−3.03 (0.02)
*Itgb1bp3*	−4.99	−80.73	−18.48	−5.26 (0.05)	−33.33 (<0.01)	−7.69 (0.04)
*Zmnd17*	−9.47	−8.79	−32.22	−4.76 (0.01)	−25.00 (0.04)	−33.33 (<0.01)
*Gbe1*	4.84	2.68	4.05	2.38 (0.05)	5.41 (<0.01)	2.81 (0.04)
*Ankrd1*	15.69	16.68	6.11	45.51 (0.04)	28.55 (<0.01)	6.81 (0.01)

All samples were compared with their WT controls to evaluate fold change. The expression of each test gene was normalized to the level of GAPDH within each sample prior to comparisons between samples. Each group represents samples from 3 mice.

Our analysis also indicates patterns of gene expression associated exclusively with polyQ AR, and maybe associated with symptoms that are found in those models but not HSA-AR (e.g. AR-immunoreactive nuclear inclusions, which are not observed in HSA-AR [41]). The list of genes in the intersection between the polyQ models (AR97Q and AR113Q) is reported in Table 2. With this comparison, we observed several previously identified candidate polyQ genes in our lists, including DnaJ (Hsp40) homolog, subfamily B, member 6 (*Dnajb6*) and P300/CBP-associated factor (*Pcaf*, Table S5). *Dnajb6*, also known as MRJ and its *drosophila* ortholog, can suppress polyglutamine toxicity and aggregate formation *in vitro* and in flies [42], [43]. The histoacetyltransferase *Pcaf* associates with polyQ AR and is found in aggregates in polyQ AR-expressing cells *in vitro* [44]. For both *Dnajb6* and *Pcaf*, the observed pattern is consistent with a protective function of altered mRNA expression within the skeletal muscle of the polyQ AR mice. We also observed similarity between our models and gene expression in skeletal muscle of a mouse model of Huntington's disease [29], although these similarities were not entirely limited to polyQ AR mice (Tables S4 and S5). This is significant, as Huntington's disease shares several similarities with KD/SBMA: it is also caused by polyglutamine expansion mutations [4], and neuromuscular pathology, including alterations in skeletal muscle gene expression are observed in mouse models of this disease [29], [45].

**Table 2:** Functional distributions of regulated genes in some clusters by Database for Annotation Visualization and Integrated Discovery (DAVID) analysis.

Pattern of Regulation	Functional Groupings	P-value
Down in all models	Protein modification, Phosphate metabolism, Endoplasmic reticulum, Regulation of neurogenesis, Glycerol metabolism, Amino acid metabolism, Calmodulin binding, Magnesium ion binding, Regulation of protein kinase activity	0.0466
Up in all models	Intracellular organelle, Transcription cofactor activity, Focal adhesion, I band	0.0481
Down in PolyQ models	Metal ion binding, Steroid metabolism, Sarcomere, Muscle contraction, Filamentous actin, Endocytosis, Chemical homeostasis, Macrophage activation, Membrane fraction	0.0408
Up in PolyQ models	Hydrolase activity, Protein binding, Peptidase activity	0.0356
Down in HSA-AR	Protein modification, Metal ion binding, Muscle contraction, Kinase activity, Neuron projection, Actin cytoskeleton, Mitochondrion, Phosphotransferase activity, Glucose metabolism	0.0563
Up in HSA-AR	Protein metabolism, I band, Response to stress, Kallikrein activity, Induction of apoptosis, Zinc ion binding, Calcium ion binding, Transcription factor binding, Actin cytoskeleton	0.0639

Functional analysis of regulated genes was performed using Functional Annotation Tool (DAVID Bioinformatics Resources) according to GO term (biological process, molecular function and cellular component) on several clusters: cluster 8 which was down in all models, cluster 15 which was up in all models, clusters 2 and 7 which were down in polyQ models only, cluster 14 which was up in polyQ models only, cluster 4 which was up only in HSA-AR and clusters 10 and 11 which were up only in HSA-AR.

Loss of AR function is thought to contribute to atrophy in KD/SBMA, as is illustrated by the advanced atrophy of polyQ AR mutant mice on a loss of AR function testicular feminization mutation (*Tfm*) genetic background [12]. The mechanism of the protective actions of AR in this regard are not currently understood, but might involve stimulation of trophic factor production [9], or activation of the Insulin-like growth factor1 (*Igf1*) pathway [40], for example. Some support for this idea was obtained in our microarray results, as we find *Vegfa* is reduced in muscle all 3 lines using qPCR (Table 1) and in the polyQ lines using microarray (Table S5). We did not find that *Igf1* was reduced, nor was the *Igf1* signaling pathway obviously dysregulated, with the exception of Insulin-like growth factor binding protein 5 (*Igfbp5*), which was decreased in AR97Q and AR113Q (Table S5). It remains possible that *Igf1* is decreased at the protein level.

**Figure 2:**
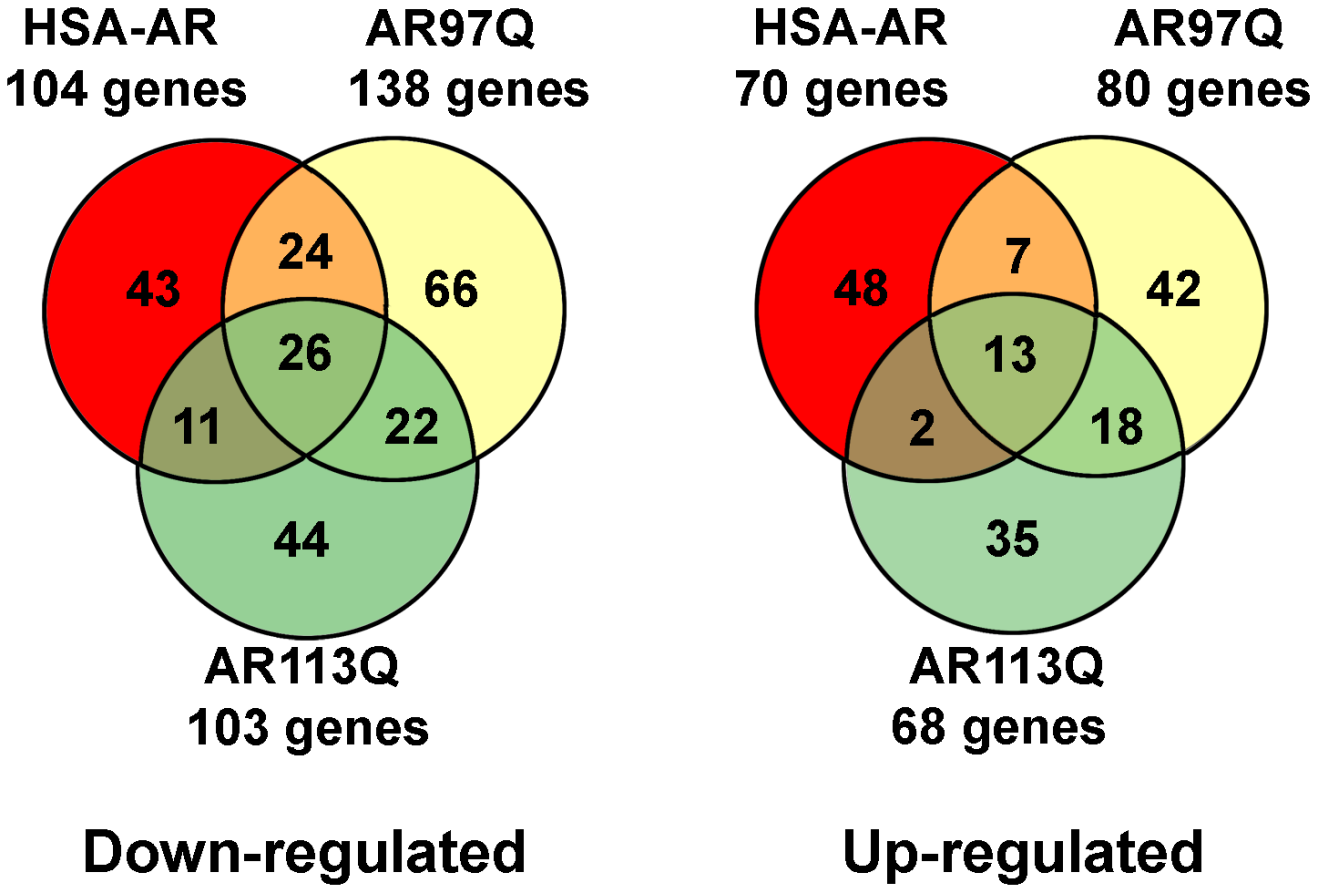
Hierarchical cluster analysis of gene expression in the 3 mouse models. Cluster output represents colorimetrically indicated log2 ratio change. Mutant strains are represented by columns and rows represent a single gene. Clusters of co-regulated genes are labeled. Most clusters are unique to one of the mutant strains. Only cluster 8 and 15 show similar patterns across the 3 models, and clusters 7 and 14 are similar in the polyQ models but not HSA-AR.

We extended this comparison of our three models by performing a hierarchical cluster analysis in an effort to further reduce the list of candidate genes (Figure 2). We found that 2 clusters of coregulated genes (cluster 8 and 15) are shared among all three models. Functional analysis of these coregulated clusters can be found in Tables 2 and 3. Analysis of gene ontogeny (GO) terms in coregulated clusters indicated diverse classes of biological functions of regulated genes, including several effects on protein metabolism, ion binding and cellular differentiation (Table 2). Interestingly, decreases in genes mediating metal ion binding were observed in both clusters unique to PolyQ models and HSA-AR, suggesting a convergence of gene function, despite different clustered genes. Kyoto encyclopedia of genes and genomes (KEGG) analysis of coregulated clusters indicated effects on the insulin signaling pathway as well as several cell adhesion and cell signaling pathways (Table 3). Insulin signaling has not previously been examined in KD/SBMA, but disturbance in mitochondria [25] could certainly result from alterations in insulin signaling [46], [47]. Similarly, alterations in cellular differentiation or tissue remodeling associated with muscle pathology could presumably account for the preponderance of these pathways.

**Table 3:** KEGG analysis of gene pathways for shared clusters.

Pathway	HSA-AR gene #	P-value	AR97Q gene #	P-value	AR113Q gene #	P-value
Insulin signaling pathway	9	0.0017	9	0.0034	4	0.1996
Focal adhesion	8	0.0420	8	0.0686	6	0.0680
Adherens Junction	4	0.1289	5	0.0500	4	0.0526
Regulation of actin cytoskeleton	6	0.2714	9	0.0398	4	0.4295
Calcium signaling pathway	7	0.0723	9	0.0159	3	
MAPK signaling pathway	2		10	0.0460	4	

Complete gene lists resulting from microarray analysis of each model were separately analyzed with DAVID and the resulting KEGG pathways and associated p-values of obtaining these pathways by chance are represented. The number of hits was determined from the gene lists manually.

Microarray results were validated using quantitative reverse transcription polymerase chain reaction (qRT-PCR) for 6 genes that were changed in all three models. Genes were chosen from microarray results to represent both increased and decreased expression and also representing a range of expression level changes. Results of qRT-PCR agreed very well with microarray results (Table 1). In each of the 18 tests performed, consistent changes in gene expression were observed. These results lead us to conclude that our microarray experiments accurately reflect the gene expression changes occurring in the different disease models.

## Discussion

This report characterizes the transcriptome of skeletal muscle in three independently generated mouse models of KD/SBMA. This study was undertaken to shed light on emerging evidence of myogenic contributions to KD/SBMA (reviewed in [5]). We were specifically interested in identifying patterns of gene expression that are shared between the 3 models, and might therefore account for the muscle wasting, motor axon loss and histopathology typical of KD/SBMA. By comparing results obtained in three models, we were able to greatly reduce the number of candidate genes.

It was surprising to find that *Fbxo32* expression was reduced in all models, as this is a prime candidate gene for muscle atrophy. However, several putative downstream effectors of *Fbxo32* were regulated similarly to other cases of muscular atrophy. Certainly there is precedent for uncoupling within the *Fbxo32* signaling pathway within myocytes [48]. One potential explanation of this observation is that preserved androgen functions in muscle repress *Fbxo32*, but that other cellular pathology initiates the pathway via alternate mechanisms. Consistent with this idea, testosterone decreases *Fbxo32* in rat levator ani muscle [49], and further loss of AR function in mouse models of KD/SBMA accelerates pathology [12]. A final possibility is that *Fbxo32* is indeed involved, but is activated by post-translational modification rather than transcriptional induction.

As we have discussed elsewhere [5], [7], it is surprising that overexpression of WT AR can reproduce some of the effects of polyQ expansion of AR. Aside from the implication that a polyQ expansion is not necessary for pathology, this observation is puzzling because polyQ AR is associated with loss of AR function, whereas overexpression of WT AR would be expected to increase AR signaling. We were therefore interested in comparing polyQ AR and HSA-AR models. We find that the overlap between HSA-AR and both AR97Q and AR113Q is comparable to the overlap between the polyQ AR models in terms of the number of genes differentially regulated in any pair-wise comparison (Figures 1 and 2). Moreover, a total of 43 genes (or 20–25% of the genes for a give model) were differentially regulated in all three models. We also examined our results for evidence of loss of AR function by comparing our gene lists with those reported for ARKO muscle [26]. As expected, several polyQ AR genes are also altered in ARKO muscle (Table S5). Surprisingly, HSA-AR samples also exhibit alterations in gene expression similar to those observed in ARKO muscle (Table S4), with gene regulation in the same direction, suggesting that a paradoxical loss of AR function may result from AR overexpression. This loss of function might result from disturbance in other components of the AR signaling pathway (e.g. titration of cofactors), or might reflect indirect effects on gene expression resulting from muscle growth, for example.

Nonetheless, some of the differentially regulated genes were shared by AR97Q and ARQ113 models but not HSA-AR (Table S5). It is tempting to speculate that this intersection includes genes that mediate aspects of KD/SBMA that are unique to polyQ expansion of AR. Notably, protein misfolding [50], aberrant proteolysis of polyQ AR [51], [52], [53] and aberrant interactions with Heat shock proteins [19], [20], [21], [23], [54], [55], [56], have all been implicated in polyQ-specific features of this disease, whereas we find no evidence of AR aggregates in HSA-AR mice [41].

One notable limitation of the current study is uncertainty concerning the androgen-dependence of the observed alterations in gene expression. Because all subjects were unmanipulated adult males, we cannot be certain that observed effects result from testosterone action, or if so, whether ongoing stimulation with testosterone is required for the alterations gene expression. We are therefore conducting experiments to evaluate this possibility by performing microarray analysis of skeletal muscle from female HSA-AR mice which are treated acutely with testosterone to induce KD/SBMA symptoms [7], [57].

## Materials and Methods

### Animals

Ten WT C57BL/6J mice (5 males and 5 females, 70 days old) from Jackson Laboratories were used to make the RNA reference samples.

Three mouse models of KD/SBMA were used in the current study: HSA-AR [7], AR113Q [6], [58], and AR97Q [59]. Two of the three models,, AR97Q and AR113Q, have a polyQ expansion in the AR. HSA-AR are transgenic (Tg) mice that overexpress selectively in skeletal muscle a rat AR with a WT number (24) of glutamine repeats [7]. AR97Q are Tg mice that express human AR under the control of a βactin/CMV promoter [59]. AR-113Q are knock-in mice in which the first exon of the endogenous mouse AR has been replaced with a similar sequence from the human AR containing 113 glutamine repeats [6].

*HSA-AR*: The production, genotyping and phenotyping of HSA-AR transgenic mice has been described elsewhere [7]. Two founding lines (L78 and L141) of HSA-AR mice, that differ in the copy number of the transgene and have corresponding differences in the severity of the neuromuscular atrophy (L178<L141) have been previously characterized. In this study, Tg male mice from L141 (n = 5) and their WT brothers (n = 8, all mice were 110–130 days of age) were used. Behavioral, histological and candidate gene expression data have been previously reported for a subset of these animals [7].

*AR113Q*: The production and genotyping of AR113Q Knock In mice has been described elsewhere [58], as has their neuromuscular phenotype [6]. Mutant males (n = 3, 3–4 months of age), fully backcrossed onto the C57BL/6J strain, were used in this study.

*AR97Q*: The production, genotyping and phenotyping of AR97Q Tg mice has been described elsewhere [59]. Tg AR97Q males and WT brothers (n = 6 of each, 112–118 days old) were used in this study.

All animal experiments conformed to NIH guidelines and were approved by the University Animal Care Committee of the University of Toronto Mississauga (Approved protocol # 20007262).

### Tissue Harvesting and Extraction of RNA

Under surgical anesthesia, limb muscles were dissected, harvested, and immediately frozen in liquid nitrogen before storage at −80°C. Frozen limb muscles were placed in TRizol Reagent (Life Technologies, NY) and homogenized before RNA extraction. The total RNA extraction was performed according to the manufacturer's guidelines. After extraction, an aliquot was used to confirm sample integrity by electrophoresis of glyoxylated RNA through 1.2% agarose gel and visualization by staining with ethidium bromide. RNA was quantified using a Nanodrop (Fisher Scientific) spectrophotometer.

### Sample Labelling, Microarray Hybridization and Data Analysis

Two-color microarray experiments were performed using 38.5K oligo mouse MEEBO arrays (Mouse Ready Arrays, Microarrays Inc., Nashville, TN). This array contains 35,302 oligonucleotide (70mer) probes is largely derived from constitutively expressed exons and represents approximately 25,000 mouse genes.

Cyanine dyes were directly incorporated into cDNA synthesized from total RNA following the procedure of Neal et al. (2003). Briefly, 38 µl reactions containing 20 µg of total RNA, 500 µmol/Lof dATP, dGTP and dTTP; 50 µmol/L dCTP (GE Life Sciences), 25 µmol/L Cy3- or Cy5-dCTP (Perkin Elmer), 10 µmol/L DTT and 150 pmol oligo dT20 primer were heated to 65°C for 5 min, then 42°C for 5 min. 2 µl SuperScript II reverse transcriptase (Invitrogen Corporation) was added, and cDNA synthesis was carried out for 3 h at 42°C. Reactions were stopped by the addition of 5 µL of 50 mmol/L EDTA. RNA was hydrolyzed with 4 µL of 5 mol/L NaOH for 10 min at 65°C, and the reaction was then neutralized by titration with acetic acid. The cDNA from one Cyanine-3 (Perkin Elmer, Boston, MA) reaction (reference sample) were combined with those from a Cyanine-5 (Perkin Elmer, Boston, MA) reaction (experiment sample) and were co-hybridized to oligo array. Images of the hybridized arrays were acquired using a ScanArray 4000 XL laser scanner (Perkin Elmer, Boston, MA) and fluorescent intensities from spots were quantified using GenePix 5 software (Axon Instruments, Inc., CA).

Microarray images and quantification data were then imported into GeneTraffic DUO (Stratagene, La Jolla, CA) for analysis. The data were normalized using the Lowess algorithm at the subgrid level while ignoring flagged values. After normalization of the data, lists of differentially expressed genes were obtained using GeneTraffic.

For the HSA-AR and AR113Q samples, a universal RNA reference sample made from C57BL/6J mice was utilized on each array. Triplicate arrays using RNA samples from the different experimental animals (i.e. HSA-AR Tg males, AR113Q Tg males,and WT brothers) were performed such that 3 animals in each mutant genotype were compared to WT controls. Log2 ratios of experimental (either mutant or their WT brothers) samples (Cy5) versus reference RNA (Cy3) were obtained. The log2 ratios from mutant samples were then subtracted from log2 ratios from WT versus reference RNA controls to find differentially regulated genes. Gene lists were filtered in GeneTraffic to include only those genes that displayed at least 2-fold difference and whose coefficient of variance was less than 100% and had a p-value less than 0.05 using a T-test.

Subsequent to the initial experiments described above, the AR97Q model became available. However, since there were not sufficient amounts of the Universal RNA reference remaining, triplicate arrays using different biological replicates of the AR97Q males (experimental, Cy5) were directly compared with their WT brothers (reference, Cy3). Dye swap experiments were not performed as previous experiments in our lab had demonstrated that they do not alter the lists of differentially expressed genes very much (Neal et. al., 2003). Using GeneTraffic, lists of genes that displayed at least 2-fold difference and whose coefficient of variance was less than 100% were made.

Hierarchical cluster analysis were performed in GeneTraffic DUO using the Pearson algorithm and average linkage [60]. Cluster figures were made using the MultiExperiment Viewer (MeV) in the TM4 suite of software tools (www.tm4.org) (Saeed et al., 2006).

All microarray data is MIAME compliant and has been deposited in GEO (accession number GSE10190).

### Real Time Quantitative RT-PCR

For the quantitative PCR (qRT-PCR) analysis, the 3 biological samples of each genotype used for the microarray analysis plus additional biological samples were used. For the HSA-AR male mice, 2 additional mice were analyzed (5 mice total). For the AR97Q and their WT controls, 3 additional mice were analyzed (6 mice total for each). For the AR113Q males, only the original 3 biological replicates were analyzed. In addition to the biological replicates, 2 or 3 technical replicates of each biological sample were performed. A two-step approach was taken in which the initial reverse transcription was followed by the qRT-PCR amplification After DNase I (Invitrogen Corporation, CA) treatment, DNA-free total RNA was reverse transcribed using a dT_20_VN primer (Sigma, Oakville, ON) with SuperScript II. Each RNA reaction had a control reaction without reverse transcriptase to evaluate any genomic DNA contamination. Two µl of the diluted reaction was used as template for each 25 µL RT-PCR amplification. Reactions were assembled using SYBR® Green JumpStart Taq ReadyMix (Sigma, Oakville, ON). The Mx4000 Multiplex Quantitative PCR System (Stratagene, La Jolla, CA) was used for data acquisition and analysis according to the instructions of the manufacturer. Samples were incubated at 95°C for 10 min prior to thermal cycling (40 cycles of: 95°C for 30 s, 57°C for 30 s, and 72°C for 30 s). In order to confirm the amplification specificity and identity the PCR products, a melting curve analysis between 55°C and 95°C was also carried out using the Mx4000 software. The completed reactions were heated to 95°C for 1 min and cooled to 55°C and reactions were re-heated in 1°C increments back to 95°C in order to plot a dissociation curve. After exporting the ROX-normalized fluorescence measurements to Microsoft Excel, the program LinRegPCR [61] was used to determine the efficiency of each reaction. These efficiencies were used in the final calculation of fold induction from the ΔC_t_ values and the expression of each test gene was normalized to the level of glyceraldehyse 3′ dehydrogenase (GAPDH) within each sample prior to comparisons between samples.

### Primer Design

The cDNA sequences for the genes, *AR*, *GAPDH*, *Fbxo32* (F-box protein 32), *Vegfa* (Vascular endothelial growth factor), *Ankrd1* (Ankyrin repeat domain 1), *Itgb1bp3* (integrin beta 1 binding protein 3), *Zmynd17* (Zinc finger, MYND domain containing 17), Gbe1 (Glucan (1,4-alpha-), branching enzyme 1), *Igf1* (Insulin-like growth factor 1), *Akt1* (Thymoma viral proto-oncogene 1) and *MuRF1* (Tripartite motif-containing 63) were obtained from public databases. PCR primers were designed from the corresponding cDNA sequences using the Whitehead Institute's Primer3 software [62]. All oligonucleotide sequences and primer pairs were checked with OligoAnalyzer 3.0 (http://scitools.idtdna.com/Analyzer/) for secondary structure and dimer formation. Each primer and amplicon sequence was tested using the nucleotide-nucleotide BLAST alignment tool to ensure minimal similarity with any other sequence. Oligonucleotide primers were obtained from Invitrogen. Primer sequences used in this study can be found in Table S6.

## Supporting Information

Table S1 Complete gene list for HSA-AR. Genes regulated in HSA-AR muscle are presented. For inclusion, each gene had to appear in the gene list generated by a 2 fold change, p≤0.05 criteria. Fold change values relative to WT controls are presented, as are Genbank accession numbers, unigene symbols and names.
(0.04 MB XLS)

Table S2 Complete gene list for AR97Q. Genes regulated in AR97Q Tg muscle are presented. For inclusion, each gene had to appear in the gene list generated by a 2 fold change, p≤0.05 criteria. Fold change values relative to WT controls are presented, as are Genbank accession numbers, unigene symbols and names.
(0.05 MB XLS)

Table S3 Complete gene list for AR113Q. Genes regulated in AR113Q muscle are presented. For inclusion, each gene had to appear in the gene list generated by a 2 fold change, p≤0.05 criteria. Fold change values relative to WT controls are presented, as are Genbank accession numbers, unigene symbols and names.
(0.04 MB XLS)

Table S4 Gene list common to all models. Genes regulated in all 3 models are presented. For inclusion, each gene had to appear in the gene list generated by a 2 fold change, p≤0.05 criteria in all models. Fold change values are presented for each model as are Genbank accession numbers, unigene symbols and names. In addition, genes that have been reported to be regulated in skeletal muscle of related conditions are identified: ARKO = genes also identified in microarray analysis of skeletal muscle of AR null mice, Atrophy = genes also identified in microarray analysis of skeletal muscle of diverse conditions resulting in muscular atrophy, DHT = genes also identified in serial analysis of gene expression (SAGE) of skeletal muscle of mice treated with dihydrotestosterone, HD = genes also identified in microarray analysis of skeletal muscle of mouse model of Huntington's disease.
(0.02 MB XLS)

Table S5 The list of up- and down-regulated genes found only in PolyQ models. Genes regulated in all 3 models are presented. For inclusion, each gene had to appear in the gene list generated by a 2 fold change, p≤0.05 criteria in AR97Q and AR113Q models. Fold change values are presented for each model as are Genbank accession numbers, unigene symbols and names. In addition, genes that have been reported to be regulated in skeletal muscle of related conditions are identified: ARKO = genes also identified in microarray analysis of skeletal muscle of AR null mice, Atrophy = genes also identified in microarray analysis of skeletal muscle of diverse conditions resulting in muscular atrophy, HD = genes also identified in microarray analysis of skeletal muscle of mouse model of Huntington's disease.
(0.02 MB XLS)

Table S6 List of primers used for quantitative RT-PCR validation of microarray results.
(0.02 MB XLS)

## References

[1] Kennedy WR, Alter M, Sung JH (1968). Progressive proximal spinal and bulbar muscular atrophy of late onset. A sex-linked recessive trait.. Neurology.

[2] Atsuta N, Watanabe H, Ito M, Banno H, Suzuki K (2006). Natural history of spinal and bulbar muscular atrophy (SBMA): a study of 223 Japanese patients.. Brain.

[3] La Spada AR, Wilson EM, Lubahn DB, Harding AE, Fischbeck KH (1991). Androgen receptor gene mutations in X-linked spinal and bulbar muscular atrophy.. Nature.

[4] Zoghbi HY, Orr HT (2000). Glutamine repeats and neurodegeneration.. Annu Rev Neurosci.

[5] Monks DA, Rao P, Mo K, Johansen JA, Lewis G (2008). Androgen receptor and Kennedy disease/spinal bulbar muscular atrophy.. Horm Behav.

[6] Yu Z, Dadgar N, Albertelli M, Gruis K, Jordan C (2006). Androgen-dependent pathology demonstrates myopathic contribution to the Kennedy disease phenotype in a mouse knock-in model.. J Clin Invest.

[7] Monks DA, Johansen JA, Mo K, Rao P, Eagleson B (2007). Overexpression of wild-type androgen receptor in muscle recapitulates polyglutamine expansion.. Proc Natl Acad Sci U S A.

[8] Katsuno M, Adachi H, Waza M, Banno H, Suzuki K (2006). Pathogenesis, animal models and therapeutics in spinal and bulbar muscular atrophy (SBMA).. Exp Neurol.

[9] Sopher BL, Thomas PS, LaFevre-Bernt MA, Holm IE, Wilke SA (2004). Androgen Receptor YAC Transgenic Mice Recapitulate SBMA Motor Neuronopathy and Implicate VEGF164 in the Motor Neuron Degeneration.. Neuron.

[10] Katsuno M, Adachi H, Sobue G (2003). Spinal and Bulbar Muscular Atrophy: Clinical Features and Pathogenesis.. ACNR.

[11] Adachi H, Waza M, Katsuno M, Tanaka F, Doyu M (2007). Pathogenesis and molecular targeted therapy of spinal and bulbar muscular atrophy.. Neuropathol Appl Neurobiol.

[12] Thomas PS, Fraley GS, Damian V, Woodke LB, Zapata F (2006). Loss of endogenous androgen receptor protein accelerates motor neuron degeneration and accentuates androgen insensitivity in a mouse model of X-linked spinal and bulbar muscular atrophy.. Hum Mol Genet.

[13] McCampbell A, Taylor JP, Taye AA, Robitschek J, Li M (2000). CREB-binding protein sequestration by expanded polyglutamine.. Hum Mol Genet.

[14] Lieberman AP, Harmison G, Strand AD, Olson JM, Fischbeck KH (2002). Altered transcriptional regulation in cells expressing the expanded polyglutamine androgen receptor.. Hum Mol Genet.

[15] Sacheck JM, Hyatt JP, Raffaello A, Jagoe RT, Roy RR (2007). Rapid disuse and denervation atrophy involve transcriptional changes similar to those of muscle wasting during systemic diseases.. FASEB J.

[16] Minamiyama M, Katsuno M, Adachi H, Waza M, Sang C (2004). Sodium butyrate ameliorates phenotypic expression in a transgenic mouse model of spinal and bulbar muscular atrophy.. Hum Mol Genet.

[17] Stenoien DL, Cummings CJ, Adams HP, Mancini MG, Patel K (1999). Polyglutamine-expanded androgen receptors form aggregates that sequester heat shock proteins, proteasome components and SRC-1, and are suppressed by the HDJ-2 chaperone.. Hum Mol Genet.

[18] Walcott JL, Merry DE (2002). Ligand promotes intranuclear inclusions in a novel cell model of spinal and bulbar muscular atrophy.. J Biol Chem.

[19] Kobayashi Y, Kume A, Li M, Doyu M, Hata M (2000). Chaperones Hsp70 and Hsp40 suppress aggregate formation and apoptosis in cultured neuronal cells expressing truncated androgen receptor protein with expanded polyglutamine tract.. J Biol Chem.

[20] Bailey CK, Andriola IF, Kampinga HH, Merry DE (2002). Molecular chaperones enhance the degradation of expanded polyglutamine repeat androgen receptor in a cellular model of spinal and bulbar muscular atrophy.. Hum Mol Genet.

[21] Ishihara K, Yamagishi N, Saito Y, Adachi H, Kobayashi Y (2003). Hsp105alpha suppresses the aggregation of truncated androgen receptor with expanded CAG repeats and cell toxicity.. J Biol Chem.

[22] Thomas M, Harrell JM, Morishima Y, Peng HM, Pratt WB (2006). Pharmacologic and genetic inhibition of hsp90-dependent trafficking reduces aggregation and promotes degradation of the expanded glutamine androgen receptor without stress protein induction.. Hum Mol Genet.

[23] Katsuno M, Sang C, Adachi H, Minamiyama M, Waza M (2005). Pharmacological induction of heat-shock proteins alleviates polyglutamine-mediated motor neuron disease.. Proc Natl Acad Sci U S A.

[24] Waza M, Adachi H, Katsuno M, Minamiyama M, Sang C (2005). 17-AAG, an Hsp90 inhibitor, ameliorates polyglutamine-mediated motor neuron degeneration.. Nat Med.

[25] Ranganathan S, Harmison GG, Meyertholen K, Pennuto M, Burnett BG (2009). Mitochondrial abnormalities in spinal and bulbar muscular atrophy.. Hum Mol Genet.

[26] MacLean HE, Chiu WS, Notini AJ, Axell AM, Davey RA (2008). Impaired skeletal muscle development and function in male, but not female, genomic androgen receptor knockout mice.. FASEB J.

[27] Yoshioka M, Boivin A, Bolduc C, St-Amand J (2007). Gender difference of androgen actions on skeletal muscle transcriptome.. J Mol Endocrinol.

[28] Yoshioka M, Boivin A, Ye P, Labrie F, St-Amand J (2006). Effects of dihydrotestosterone on skeletal muscle transcriptome in mice measured by serial analysis of gene expression.. J Mol Endocrinol.

[29] Strand AD, Aragaki AK, Shaw D, Bird T, Holton J (2005). Gene expression in Huntington's disease skeletal muscle: a potential biomarker.. Hum Mol Genet.

[30] Lecker SH, Jagoe RT, Gilbert A, Gomes M, Baracos V (2004). Multiple types of skeletal muscle atrophy involve a common program of changes in gene expression.. Faseb J.

[31] Miyazaki M, Esser KA (2009). REDD2 is enriched in skeletal muscle and inhibits mTOR signaling in response to leucine and stretch.. Am J Physiol Cell Physiol.

[32] Pisani DF, Leclerc L, Jarretou G, Marini JF, Dechesne CA (2005). SMHS1 is involved in oxidative/glycolytic-energy metabolism balance of muscle fibers.. Biochem Biophys Res Commun.

[33] Eigenthaler M, Engelhardt S, Schinke B, Kobsar A, Schmitteckert E (2003). Disruption of cardiac Ena-VASP protein localization in intercalated disks causes dilated cardiomyopathy.. Am J Physiol Heart Circ Physiol.

[34] Li J, Rao H, Burkin D, Kaufman SJ, Wu C (2003). The muscle integrin binding protein (MIBP) interacts with alpha7beta1 integrin and regulates cell adhesion and laminin matrix deposition.. Dev Biol.

[35] Li J, Mayne R, Wu C (1999). A novel muscle-specific beta 1 integrin binding protein (MIBP) that modulates myogenic differentiation.. J Cell Biol.

[36] Wootton PT, Flavell DM, Montgomery HE, World M, Humphries SE (2007). Lipoprotein-associated phospholipase A2 A379V variant is associated with body composition changes in response to exercise training.. Nutr Metab Cardiovasc Dis.

[37] Fernando SM, Rao P, Niel L, Chatterjee D, Stagljar M (2010). Myocyte androgen receptors increase metabolic rate and improve body composition by reducing fat mass.. Endocrinology.

[38] Orngreen MC, Schelhaas HJ, Jeppesen TD, Akman HO, Wevers RA (2008). Is muscle glycogenolysis impaired in X-linked phosphorylase b kinase deficiency?. Neurology.

[39] Wuyts W, Reyniers E, Ceuterick C, Storm K, de Barsy T (2005). Myopathy and phosphorylase kinase deficiency caused by a mutation in the PHKA1 gene.. Am J Med Genet A.

[40] Palazzolo I, Burnett BG, Young JE, Brenne PL, La Spada AR (2007). Akt blocks ligand binding and protects against expanded polyglutamine androgen receptor toxicity.. Hum Mol Genet.

[41] Monks DA, Johansen JA, Mo K, Rao P, Eagleson B (2007). Overexpression of wild-type androgen receptor in muscle recapitulates polyglutamine disease.. Proc Natl Acad Sci U S A.

[42] Chuang JZ, Zhou H, Zhu M, Li SH, Li XJ (2002). Characterization of a brain-enriched chaperone, MRJ, that inhibits Huntingtin aggregation and toxicity independently.. J Biol Chem.

[43] Fayazi Z, Ghosh S, Marion S, Bao X, Shero M (2006). A Drosophila ortholog of the human MRJ modulates polyglutamine toxicity and aggregation.. Neurobiol Dis.

[44] Katsuno M, Adachi H, Minamiyama M, Waza M, Doi H Disrupted transforming growth factor-beta signaling in spinal and bulbar muscular atrophy.. J Neurosci.

[45] Ribchester RR, Thomson D, Wood NI, Hinks T, Gillingwater TH (2004). Progressive abnormalities in skeletal muscle and neuromuscular junctions of transgenic mice expressing the Huntington's disease mutation.. Eur J Neurosci.

[46] Boirie Y, Short KR, Ahlman B, Charlton M, Nair KS (2001). Tissue-specific regulation of mitochondrial and cytoplasmic protein synthesis rates by insulin.. Diabetes.

[47] Stump CS, Short KR, Bigelow ML, Schimke JM, Nair KS (2003). Effect of insulin on human skeletal muscle mitochondrial ATP production, protein synthesis, and mRNA transcripts.. Proc Natl Acad Sci U S A.

[48] Tong JF, Yan X, Zhu MJ, Du M (2009). AMP-activated protein kinase enhances the expression of muscle-specific ubiquitin ligases despite its activation of IGF-1/Akt signaling in C2C12 myotubes.. J Cell Biochem.

[49] Pires-Oliveira M, Maragno AL, Parreiras-e-Silva LT, Chiavegatti T, Gomes MD Testosterone represses ubiquitin ligases atrogin-1 and Murf-1 expression in an androgen-sensitive rat skeletal muscle in vivo.. J Appl Physiol.

[50] Thomas M, Yu Z, Dadgar N, Varambally S, Yu J (2005). The unfolded protein response modulates toxicity of the expanded glutamine androgen receptor.. J Biol Chem.

[51] Merry DE, Kobayashi Y, Bailey CK, Taye AA, Fischbeck KH (1998). Cleavage, aggregation and toxicity of the expanded androgen receptor in spinal and bulbar muscular atrophy.. Hum Mol Genet.

[52] Li M, Chevalier-Larsen ES, Merry DE, Diamond MI (2007). Soluble androgen receptor oligomers underlie pathology in a mouse model of spinobulbar muscular atrophy.. J Biol Chem.

[53] Mandrusiak LM, Beitel LK, Wang X, Scanlon TC, Chevalier-Larsen E (2003). Transglutaminase potentiates ligand-dependent proteasome dysfunction induced by polyglutamine-expanded androgen receptor.. Hum Mol Genet.

[54] Adachi H, Katsuno M, Minamiyama M, Sang C, Pagoulatos G (2003). Heat shock protein 70 chaperone overexpression ameliorates phenotypes of the spinal and bulbar muscular atrophy transgenic mouse model by reducing nuclear-localized mutant androgen receptor protein.. J Neurosci.

[55] Wyttenbach A, Carmichael J, Swartz J, Furlong RA, Narain Y (2000). Effects of heat shock, heat shock protein 40 (HDJ-2), and proteasome inhibition on protein aggregation in cellular models of Huntington's disease.. Proc Natl Acad Sci U S A.

[56] Adachi H, Waza M, Tokui K, Katsuno M, Minamiyama M (2007). CHIP overexpression reduces mutant androgen receptor protein and ameliorates phenotypes of the spinal and bulbar muscular atrophy transgenic mouse model.. J Neurosci.

[57] Johansen JA, Yu Z, Mo K, Monks DA, Lieberman AP (2008). Recovery of function in a myogenic mouse model of spinal bulbar muscular atrophy.. Neurobiol Dis.

[58] Yu Z, Dadgar N, Albertelli M, Scheller A, Albin RL (2006). Abnormalities of germ cell maturation and sertoli cell cytoskeleton in androgen receptor 113 CAG knock-in mice reveal toxic effects of the mutant protein.. Am J Pathol.

[59] Katsuno M, Adachi H, Kume A, Li M, Nakagomi Y (2002). Testosterone reduction prevents phenotypic expression in a transgenic mouse model of spinal and bulbar muscular atrophy.. Neuron.

[60] Eisen MB, Spellman PT, Brown PO, Botstein D (1998). Cluster analysis and display of genome-wide expression patterns.. Proc Natl Acad Sci U S A.

[61] Ramakers C, Ruijter JM, Deprez RH, Moorman AF (2003). Assumption-free analysis of quantitative real-time polymerase chain reaction (PCR) data.. Neurosci Lett.

[62] Rozen S, Skaletsky H (2000). Primer3 on the WWW for general users and for biologist programmers.. Methods Mol Biol.

